# Salp15, a Multifunctional Protein From Tick Saliva With Potential Pharmaceutical Effects

**DOI:** 10.3389/fimmu.2019.03067

**Published:** 2020-01-10

**Authors:** Shiyuan Wen, Feng Wang, Zhenhua Ji, YingYi Pan, Miaomiao Jian, YunFeng Bi, Guozhong Zhou, Lisha Luo, Taigui Chen, Lianbao Li, Zhe Ding, Manzama-Esso Abi, Aihua Liu, Fukai Bao

**Affiliations:** ^1^Department of Microbiology and Immunology, Kunming Medical University, Kunming, China; ^2^The Center of Tropical Diseases, The Institute for Tropical Medicine, Kunming Medical University, Kunming, China; ^3^Yunnan Demonstration Base of International Science and Technology Cooperation for Tropical Diseases, Kunming, China; ^4^The First People's Hospital of Yunnan Province, The Affiliated Hospital of Kunming University of Science and Technology, Kunming, China; ^5^Department of Biochemistry and Molecular Biology, Kunming Medical University, Kunming, China

**Keywords:** tick, Salp15, *Borrelia burgdorferi*, T cell, immunomodulation, therapeutic effects

## Abstract

*Ixodes* ticks are the main vectors for a number of zoonotic diseases, including Lyme disease. Ticks secrete saliva directly into a mammalian host while feeding on the host's blood. This action serves to modulate host immunity and coagulation, thus allowing ticks to attach and feed upon their host. One of the most extensively studied components of tick saliva is Salp15. Research has shown that this protein binds specifically to CD4 molecules on the surface of T lymphocytes, interferes with TCR-mediated signaling transduction, inhibits CD4+ T cell activation and proliferation, and impedes the secretion of interleukin 2 (IL-2). Salp15 also binds specifically to dendritic cell dendritic cell-specific intercellular adhesion molecule-3-grabbing non-integrin (DC-SIGN) to up-regulate the expression of CD73 in regulatory T cells. Collectively, these findings render this salivary protein a potential candidate for a range of therapeutic applications. Here, we discuss our current understanding of Salp15 and the mechanisms that might be used to treat disease.

## Introduction

Mosquitoes and ticks are considered the most predominant vectors for the transmission of various pathogens to humans and animals (1, 2). Lyme disease is one of the most common tick-borne diseases worldwide. It is caused by an infection with *Borrelia burgdorferi* sensu lato (*B. burgdorferi* s.l.), and was first discovered in the United States in the mid-1970s. Burgdorfer et al. (1982) were the first to characterize and isolate the causative agent of Lyme disease from *Ixodes scapularis* (*I. scapularis*). Those authors demonstrated that *B. burgdorferi*, a bacterial species of the spirochete class, was responsible for the majority of infections and was mostly detected in the midgut of ticks (3).

In order to avoid host defenses during a blood meal, *Ixodes* ticks secrete a cocktail of bioactive factors in their saliva, including immunomodulatory molecules, gasket and holdfast elements, wound healing inhibitors, analgesic factors, vasoconstriction mediators, anti-hemostatic and anti-inflammatory factors (4–17). These multi-function components have potential applications in the treatment of disease. Das et al. (18) previously reported interesting findings relating to antigens in the tick salivary gland. That research led to the first identification of a 15-kDa salivary protein in *I. scapularis*, which was named Salp15 after its calculated molecular mass. Subsequent studies showed that Salp15 inhibits the proliferation of CD4+ T cells and reduces the production of cytokines such as IL-2, in a dose-dependent manner. These data indicate that Salp15 exhibits properties that suppress the immune system of hosts (4). In addition, Salp15 has been shown to interact with the C-type lectin receptor, DC-SIGN, resulting in activation of the RAF-1/MEK-dependent signaling pathway; this impairs cytokine production and T cell proliferation (19, 20).

Collectively these earlier findings indicated that Salp15 has potential as a promising therapeutic candidate for a variety of clinical applications. Indeed, Paveglio et al. (21) reported that Salp15 effectively suppresses the immune response in a mouse model of allergic asthma by binding and inhibiting CD4+ T cells. More recent research has also revealed the role of Salp15 in the pathogenesis of a range of diseases; these important findings are discussed below. Over the past two decades, an increasing number of research studies have attempted to identify and characterize Salp15 homologs in various *Ixodes* species (22–25). The immunomodulatory effects of Salp15 present important opportunities for the development of novel and sophisticated therapies for human disease. However, little is known about the specific role of Salp15 in autoimmune diseases. This is probably because of a general lack of recognition of the potential importance of this particular protein, which the present review aims to address. In this review, we describe our current understanding of Salp15 and discuss its role in pathogen-vector-host interactions. In particular, we discuss the mechanisms underlying the immunosuppressive effect induced by the interaction of Salp15 with the host and the capacity of this protein to regulate the immune system in a range of diseases, including asthma and hematopoietic transplantation. We also discuss the potential applications of Salp15 as an attractive candidate for immunotherapy.

## Identification of Salp15 and Its Homologs

In order to confirm the identity of the specific antigen from the tick salivary gland that can initiate an antibody-mediated immune response in a host, Das et al. (18) acquired serum from *I. scapularis*-immune rabbits and carried out an immunoscreen with 100,000 clones of a tick salivary gland expression cDNA library. These authors successfully identified 47 clones, which exhibited specific reactions; these clones encoded 14 genes. One of the products of these 14 genes, a 14.7 kDa basic protein, is expressed when ticks feed on the blood of a host. This salivary protein is encoded by a 408-base pair gene with a 20-amino acid signal peptide, has an isoelectric point of 9.7, and was named Salp15 (18). Salp15 has become one of the most widely studied salivary proteins from ticks and is a cysteine-rich glycosylation protein (26). Previous studies synthesized a recombinant version of Salp15 from transfected *Drosophila melanogaster* S2 cells to facilitate further research (26). More recently, however, studies have more commonly utilized *Escherichia coli* as an expression system for Salp15, as this system is not only easy to handle, but also achieves considerable yields and good solubility; these attributes are of significance in the practical application of Salp15 in anti-tick vaccines (26, 27).

Homologs of Salp15 have been identified in other *Ixodes* species (22–25, 28–32). We searched a protein database using online software (National Center for Biotechnology Information, NCBI) for proteins from *I. scapularis* that are similar to Salp15. We successfully downloaded amino acid sequences of homologs to Salp15 from *I. sinensis* (five sequences), *I. scapularis* (17 sequences), *I. ricinus* (18 sequences), *I. persulcatus* (12 sequences), *I. pacificus* (two sequences), *I. holocyclus* (seven sequences) and *I. affinis* (one sequence).

**Figure 1:**
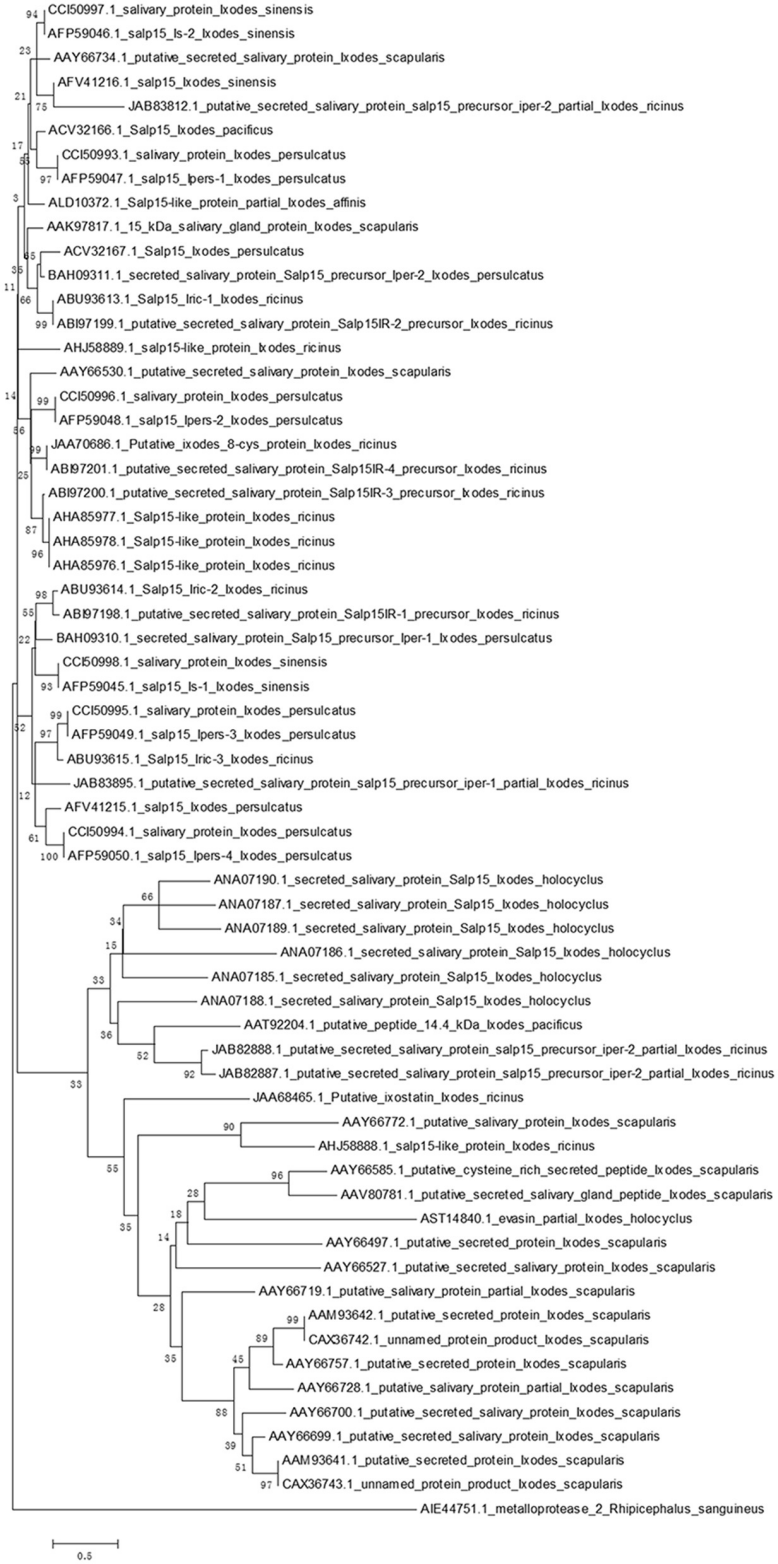
Phylogenetic analysis of Salp15 protein family. A phylogenetic tree of Salp15 homologs was generated using amino acid sequences from *I. scapularis, I. pacificus, I. ricinus, I. persulcatus, I. holocyclus*, and *I. sinensis*. Accession numbers for the sequences are shown. Sequences were aligned using ClustalW software online, and the phylogenetic tree was constructed using MEGA 5 software.

In order to create a stable phylogenetic tree, we then selected metalloprotease 2, a salivary protein from *Rhipicephalus sanguineus*, as an outgroup. GenBank accession numbers for these sequences are shown in Figure 1. Sequences were aligned using ClustalW (33). A rooted neighbor-joining tree was then constructed with the Mega 5.0 software (34). Bootstrap sampling was reiterated 1,000 times (22, 32). The phylogenetic tree clearly showed that the Salp15 family is conserved across a range of *Ixodes* species (Figure 1). Furthermore, the amino acid sequence of Salp15 remained homogeneous among various species during evolution (Figure 1). The Salp15 family also showed conservation across different protein families (Figure 1). Other studies have shown that the Salp15 protein family has undergone a phase of adaptive evolution (35). Indeed, the inter-species and intra-species similarities of Salp15 are quite close (32). A recent study used bioinformatics analysis to predict post-translational modifications of Salp15 and its homologs; the results suggested that all Salp15 family members contain at least two N-linked glycosylation sites (25). Analysis of our phylogenetic tree provided further support for these earlier findings. Thus far, studies investigating the conservation of Salp15 homologs in *I. persulcatus, I. ricinus*, and *I. pacificus* have been mainly confined to the C-terminus; this is because this site specifically interacts with CD4 molecules on T cells (22, 30, 31). Studies have confirmed that Salp15 from *I. persulcatus* can bind with *Borrelia* outer surface proteins C (OspCs) to protect the spirochetes from antibody-mediated killing, as well as phagocytosis, and its homolog derived from *I. ricinus* exhibits immunomodulatory effects on the host (23, 24, 29).

## Salp15 Mediates Relationships in the Pathogen-Tick-Host Triangle

*Ixodes* ticks belong to the Ixodidae family, which are obligate ectoparasites and can transmit a variety of pathogens to a host while feeding on mammalian blood. The developmental life cycle of *Ixodes* consists of four stages: eggs, larvae, nymphs, and adults (36). *Ixodes* eggs hatch into larvae under suitable conditions; ticks must feed on blood to enable the larvae to enter the next developmental phase. There may be one, two, or three hosts throughout the life cycle; the precise number depends on the species of tick (37, 38). Ticks become infected with tick-borne pathogens while feeding on a competent reservoir, and transmit these pathogens to new vertebrate hosts during subsequent blood feeding (39). Among several zoonotic diseases, Lyme disease is a major issue in North America and parts of Europe and Asia, as it poses significant threats to public health and the economy (40–42). However, the vectors responsible for Lyme disease vary widely according to the geographic location. For example, in the eastern and upper midwestern regions of the United States, *I. scapularis* is known to be the main vector responsible for Lyme disease; while, the predominant vectors in the western/northern and northwestern regions are *I. pacificus* and *I. affinis*, respectively (43, 44). In contrast, in Europe and Eurasia, *B. burgdorferi* s.l. is transmitted by *I. ricinus* and *I. persulcatus* (45, 46). According to recent reports, the *B. burgdorferi* s.l. complex consists of more than 20 members, including *B. burgdorferi* sensu stricto, *B. garinii*, and *B. afzelii*; collectively, these represent the main causative agents of human Lyme disease (47–49). *B. burgdorferi* s.l. could survive in the human body for a long period time, causing extensive damage to several organs and systems, including the skin, joints, heart, and nervous system. The clinical manifestations of Lyme disease are diverse and non-specific, but are generally divided into the early, middle, and late stages. The early stage is characterized by erythema migrans (50). Approximately 5% of untreated patients will develop neuroborreliosis and cardiac dysfunction. The late stage is characterized by arthritis, which can lead to disability or even death (50).

*B. burgdorferi* s.l. has developed the ability to utilize secreted tick saliva to facilitate its colonization of a mammalian host (20, 51). The genome of *B. burgdorferi* s.l. contains at least 1.4 × 10^6^ base pairs. The majority of these genes encode lipoproteins; almost all of the Osps of *B. burgdorferi* s.l. are typical bacterial lipoproteins (52). In a previous study, Pal et al. (53) identified the tick receptor for OspA (TROSPA) in the gut of *I. scapularis*, which binds specifically to OspA in *B. burgdorferi*; this binding reaction is essential for the colonization of pathogens with ticks. Those authors also reported a significant increase in the expression of TROSPA when *B. burgdorferi* infects ticks. Once a tick is engorged after feeding, the expression level of TROSPA decreases and OspA is downregulated (53, 54). During the life cycle of the tick-host interaction, the spirochetes living within the ticks can alter the structure of their outer surface to successfully propagate and transmit the pathogen to another host. When *B. burgdorferi* infects ticks, the expression OspA is upregulated; this facilitates spirochete replication within the tick gut (53). However, while feeding, *B. burgdorferi* causes a downregulation of OspA and an upregulation of OspC in the gut; during this period, *B. burgdorferi* can be transferred from the gut to the tick salivary glands, and then be transmitted to the host via the tick saliva (55). In addition, Pal et al. (56) revealed that OspC can bind with the *I. scapularis* salivary gland in a strong and specific manner, and that OspC is critical for invasion of the salivary glands during transmission. The capacity of OspC-deficient *B. burgdorferi* to be transmitted into mice was ~800-fold lower than that of normal spirochetes. The composition of tick salivary is complex and changes during the blood meal (16, 57). Research has revealed that Salp15, a salivary gland protein, could bind specifically with OspC in *B. burgdorferi*, both *in vitro* and *in vivo*. Moreover, the expression of Salp15 was selectively enhanced in the salivary glands of *B. burgdorferi*-infected *I. scapularis* during blood feeding (56). In another study (51), the inhibition of Salp15 expression by RNA interference significantly reduced the capacity for *B. burgdorferi* transmission to mice. The binding of Salp15 with *B. burgdorferi*, and the increased expression of Salp15 induced by *B. burgdorferi* in engorged ticks are specific; consequently, Salp15 is not enhanced in the tick salivary gland in response to other tick-borne pathogens, such as *Anaplasma phagocytophilum* (51, 58).

*B. burgdorferi* can stimulate specific and non-specific immune responses in the host, particularly humoral immunity against spirochetes. Furthermore, OspC plays a vital role in establishing initial infection in the host by the invasion of pathogens (56, 59). Ramamoorthi et al. (51) reported that Salp15 can bind to OspC on the surface of *B. burgdorferi* and protect spirochetes from antibody-mediated killing. Interestingly, the protective effect of Salp15 on spirochetes begins to weaken after the first 24 h. During this time, Salp15 could facilitate spirochete to reproduction within the host. Further research has shown that Salp15 also helps spirochetes colonization in mice that had been previously exposed to *B. burgdorferi*. The complement system is not only an important component of the innate immune defense system but is also involved in adaptive immunity. *B. burgdorferi* sensu stricto, *B. garinii*, and *B. afzelii* can activate complement in normal human plasma (NHS) by the alternative pathway or classical pathway, and spirochetes can be killed by the membrane attack complex (MAC) formed by the activated complement (60). One study demonstrated that *B. burgdorferi* sensu stricto, *B. garinii*, and *B. afzelii*, with specific adherence of Salp15 to OspC on their surfaces, are protected from complement-mediated killing. Salp15 also hampers the deposition of the C5b-9 complement complex on the spirochete membrane, thereby preventing the formation of the MAC and suspending the terminal effect of complement activation (29).

The interaction between Salp15, which is one of the potent immunosuppressive agents, and other bioactive tick saliva components could facilitate the prolonged feeding of ticks on the vertebrate host. Owing to the consequent increase in the length of attachment, the host initiates an immune response against the vector. However, ticks also secrete saliva to regulate host immunity and ensure that they can feed successfully. The longer the attachment time, the better the transmission of pathogens. Salp15 can be readily detected at the site of natural inoculation in tick-infested mice (4). The precise impact of Salp15 on the host will be described later in this review. The triangulated form of interaction between the pathogen-vector, vector-host, and pathogen-host, provides favorable conditions for *Borrelia* survival and transmission. When considering the interactions between OspC and spirochetes, the already complex web of interactions has evidently undergone a period of adaptive evolution (35).

## Salp15 Inhibits Immune Function in Mammalian Hosts

### Salp15 Inhibits the Activation of CD4+ T Cells

**Figure 2:**
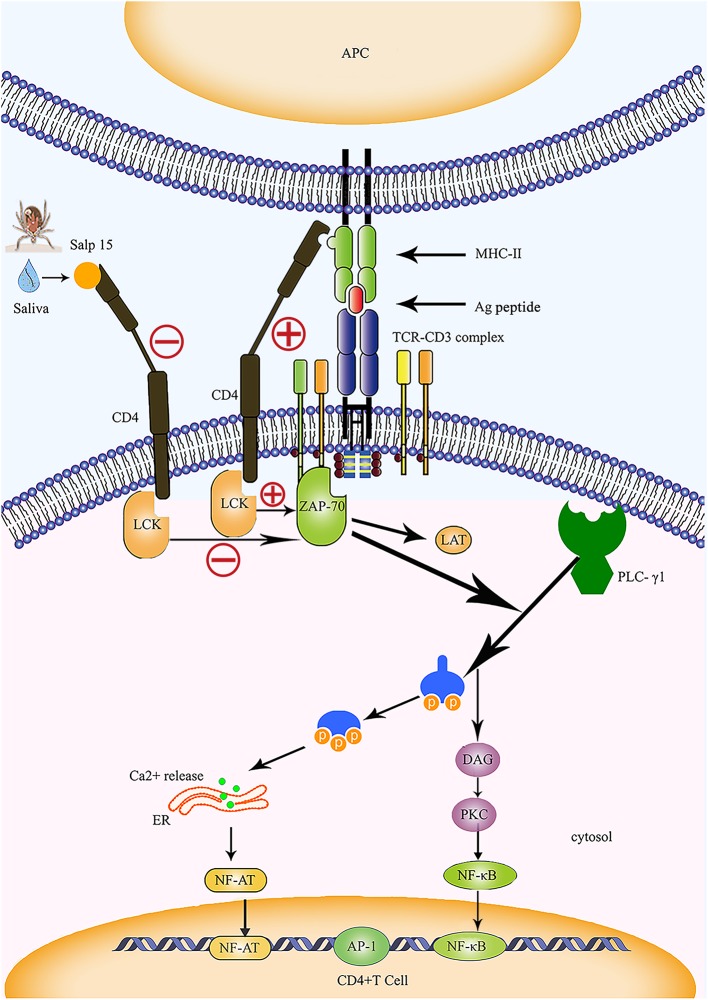
Salp15 specifically binds to CD4 molecules on the surface of T lymphocytes, thus inhibiting TCR-mediated signal transduction. Under normal circumstances, CD4 binds to the MHC-II molecule and TCR binds to the peptide created when the TCR complex interacts with the peptide-MHC complex; this activates LCK and initiates a biochemical cascade reaction. Binding between Salp15 and CD4 can impede the activation of LCK during the activation of CD4+ T cells. During the activation of CD4+ T cells, Salp15, and CD4 binding inhibits LCK activation and prevents the activation of a series of downstream substrates, including ZAP70, LAT, and PLC-γ1. Eventually, there is a significant reduction in the intracellular calcium flux when CD4+ T cells are activated, which reduces the binding ability of NF-AT and NF-κB to DNA and impairs the production of IL-2. However, AP-1 transcriptional activity and other non-TCR-mediated signaling events are not affected by Salp15. ER, endoplasmic reticulum; LAT, linker of activation in T cells.

In humans and animals, stimulation by antigens results in the transformation of T lymphocytes into effector T cells. This causes the release of a variety of cytokines which induce specific immune responses. The effect of inflammatory responses mediated by CD4+ T cells plays an important role in anti-intracellular parasitic pathogen infection. Das et al. (4, 18) were the first to identify Salp15 in tick saliva, and showed that this protein exhibits partial homology with the two active motif regions of inhibin A, a member of the transforming growth factor beta (TGF-β) superfamily. The TGF-β impedes the proliferation of T cells and the production of cytokines, suggesting that Salp15 may also exhibit immunomodulatory properties. In 2002, Anguita et al. (4) reported that Salp15 inhibited the activation of CD4+ T cells and the production of IL-2. Normally, the T cell receptor (TCR) and CD3 form a complex by forming non-covalent bonds on the surface of T cells; in this reaction, TCR is responsible for recognizing the antigen peptide, while CD3 is responsible for transmitting TCR-mediated extracellular signals to the interior of the cell (Figure 2). The cell membrane stimulation signal is converted into a state of cell activation via a signal transduction pathway. The specific interaction and binding of peptide-MHC complexes on the surface of the antigen-presenting cell (APC) to CD4 and TCR on the surface of T cells are essential for the activation of naive T cells (Figure 2). Data from several studies suggest that Salp15 binds to CD4 molecules on the surface of CD4+ T cells, and Salp15 exerts an inhibitory effect on the activation of CD4+ T cells by interfering with TCR-mediated signaling (4, 61). *In vitro* experiments further reveal that Salp15 only inhibits the activation of naive CD4+ T cells and have no effect on the activation of effector CD4+ T cells (4). These results led to the hypothesis that drugs targeting CD4+ T cells might be safer than non-specific immunosuppressive agents.

#### Salp15 Exhibits Specific Interaction With CD4

Further study revealed that Salp15 can specifically bind to CD4 molecules on the surfaces of T cells (Figure 2). Garg et al. (62) reported the specific immunoprecipitation of His-tagged Salp15 with CD4 and showed that Salp15 also binds to non-lymphocytes expressing CD4 molecules. This indicates that CD4 and Salp15 can interact in both a direct and specific manner. Monomeric Salp15 can interact with the soluble ectodomains (D1-2 and D1-4) of the CD4 receptor, forming 1:1 Salp15·soluble CD4 (sCD4) D1–D2 and Salp15·sCD4D1–D4 complexes (63). The 20-amino acid C-terminal residues of Salp15 (P11) interact specifically with the most extracellular domain (D1) of CD4 (Figure 2). Salp15 is also a specific ligand for the CD4 co-receptor; this binding induces structural rearrangement of the CD4 receptor on the long axis. As a result of the interaction with Salp15, sCD4D1–D2 and sCD4D1–D4 alter the structure of the initial bilobal and tetralobal structures, respectively (63). Architectural changes in the four extracellular domains of the CD4 molecule result in a myriad of signaling changes within T cells (62, 63). Furthermore, the specific interaction of Salp15 with CD4 causes defects in actin polymerization and a reduction in lipid raft clustering; this has significant consequences for the amplification and transduction of TCR-mediated signals during the activation of T cells (4, 64).

Other research has shown that Salp15 can bind to CD4 for extended periods of time and exert immunosuppressive effects. Purified splenic CD4+T cells, after being stimulated in the presence of Salp15 for 2 days, were then extensively washed and re-stimulated for another 2 days; the inhibitory effects of Salp15 on T cells were still observed, even after removing contact with salivary proteins after 4 days of activation (65). Furthermore, flow cytometry was used to detect the binding of Salp15 to CD4 for up to 72 h; the results suggested that the binding reaction between CD4 and salivary protein is persistent (65). However, deletion of the salivary protein C-terminal peptide P11 (Salp15ΔP11) results in a significant reduction in the capacity of Salp15 to bind to CD4+ T cells and a consequential lack of biological activity (65, 66). Understanding the function and conformation of Salp15 can facilitate the development of highly specific pharmacological agents, such as monoclonal antibodies (mAbs) or Salp 15-like peptides, for special uses.

#### Salp15 Inhibits the TCR-Mediated Signaling Pathway

The CD4 molecule is a glycoprotein expressed on the surfaces of mature T cells that recognizes MHC class II molecules, plays a role in enhancing the interaction between T cells and APC, assists TCR in recognizing the antigen, and is considered a co-receptor of TCR. The CD4 molecule possesses a cytoplasmic tail that can be associated with tyrosine kinase p56lck; activation of p56lck can phosphorylate immunoreceptor tyrosine-based activation motifs in the intracellular region of CD3 and facilitate the activation of T cells (67) (Figure 2). Usually, recognition and binding of the peptide-MHC complex to TCR and CD4 occurs on the surfaces of the APCs and CD4+ T cells, and represents one of the essential signals for the activation of naive T cells. This signal is delivered to the cell by CD3 and results in a biochemical cascade reaction (Figure 2). The specific binding of Salp15 to CD4 impairs the capacity for CD4- p56lck interactions; however, this effect only occurs in stimulated CD4+ T cells and is abrogated in unstimulated cells (4, 63). Under normal circumstances, activated zeta-chain-associated protein kinase 70 (ZAP 70) synergizes with multiple kinases to cause enzyme-active phospholipase C-γ1 (PLC-γ1) to cleave phosphatidylinositol 4,5-bisphosphate (PIP2) into two vital signaling molecules, inositol 1,4,5 phosphate (IP3) and diacylglycerol (DAG), both of which activate different downstream signaling pathways (68–70) (Figure 2).

Garg et al. (62) were the first to report the interaction between Salp15 and T cell co-receptors, revealing that Salp15 suppresses the activation of LCK by preventing the dephosphorylation of tyrosine and reducing the levels of tyrosine phosphorylation at different positions on LCK. The incubation of CD4+ T cells with Salp15 also impairs the phosphorylation of ZAP-70. This indicates that the inhibitory effect of Salp15 on the activation of CD4+ T cells does not occur by interference in the interaction between MHC II and CD4, but by inhibition of TCR-mediated signaling, which is essential for T cell activation (62) (Figure 2). Furthermore, the effect of Salp15 on TCR signaling impedes the phosphorylation of PLC-γ1 downstream and ultimately leads to a significant reduction in calcium flux during the activation of CD4+ T cells (62). These results are also supported by another investigation, which demonstrated that Salp15 reduces the tyrosine phosphorylation of several proteins in the upstream signaling pathway during PLC-γ1 activation (64). In addition to LCK and ZAP-70, Vav1, Lat, and CD3ξ are all known to be affected by Salp15, which can cause a specific reduction in the levels of tyrosine phosphorylation among these signaling proteins. Moreover, Salp15 is not species-specific with regards to its interference with TCR signaling (64).

Salp 15 is a promising candidate with potential applications as a pharmaceutical agent for pathologies mediated by CD4+ T cells. Rheumatoid arthritis (RA) is a chronic autoimmune disease characterized by synovial joint inflammation and progressive destruction of cartilage and bone (71). The cytokine signaling system is closely related to the pathogenesis of rheumatoid arthritis, when T cells are activated and CD4+ T cells infiltrate the synovium of patients with RA (72, 73). This means that targeted regulation of CD4+ T cells may play an important role in the treatment of RA (74, 75). Although no experimental evidence currently exists, the interference of Salp15 in CD4+T cell signaling may facilitate the discovery of a new generation of RA treatments. Furthermore, Salp15 modulates host immune cells by interacting with CD4 molecules and could potentially have therapeutic applications in T cell-mediated autoimmune diseases.

#### Salp 15 Inhibits the Production of IL-2

As one of the first cytokines to be discovered, IL-2 is mainly produced by activated CD4+ T cells. It plays a key role in the growth and differentiation of T cells (74). Regulatory elements are known to exist within the proximal IL-2 promoter, including nuclear factor (NF)-κB, NF-AT, and activator protein (AP)-1. We previously mentioned that Salp15 inhibits T cell signaling pathways, thus affecting Ca2+ mobilization and TCR-mediated transcriptional activation (4, 64). Salp15 also significantly reduces NF-AT and NF-κB DNA binding capacity in CD4+ T cells, resulting in the inhibition of IL-2 production (Figure 2). However, AP-1 transcriptional activity and other non-TCR-mediated signaling events are not affected by Salp15 (62, 64).

The levels of IL-2 produced by stimulated CD4+ T cells are markedly reduced in the presence of Salp15; furthermore, IL-2 production can be inhibited at both the cytokine and gene levels (4, 65). In the presence of Salp15, a significant reduction in *IL-2* mRNA levels can be detected in activated CD4+ T cells (4). Transcriptomic analysis reveals that in the early stages of CD4+ T cell activation, Salp15 reduces the expression of certain genes, including *Il2* and *cd44* (65). The inhibition of IL-2 production by tick saliva completely disappears following the addition of Salp15 antisera; furthermore, Salp15 inhibits the production of IL-2 by CD4+ T cells in a dose-dependent manner (4). When activated, CD4+T cells express the alpha chain of the IL-2 receptor (CD25), which is pivotal for CD4+T cell activation. Although the inhibition of *Il2ra* expression by Salp15 is not significant at the gene level, further analysis shows a marked reduction in CD25 expression on the surfaces of CD4+T cells, and this inhibitory effect persists throughout the activation phase of CD4+ T cells (4, 65). It is interesting to note that this suppressive effect of Salp15 is more pronounced when lower concentrations of anti-CD3 are used for stimulation or when costimulatory molecules are lacking. These data imply that Salp15 interferes with early TCR-mediated CD4+T cell activation (4). The inhibitory effect of Salp15 on CD4+ T cells is long-lasting, as noted earlier in this review. In previous research, after purified splenic CD4+T cells were stimulated in the presence of Salp15 for 2 days and then extensively washed and re-stimulated for another 2 days, IL-2 levels were significantly reduced at both time points (65). Consequently, the evidence seems to indicate that Salp15 inhibits the activation of CD4+ T cells by impairing the production of IL-2. These findings show that Salp15 inhibits the production of IL-2. Thus, Salp15 may be a therapeutic candidate to suppress pathologies induced by IL-2 overexpression. For example, in psoriasis, a long-lasting autoimmune disease characterized by patches of abnormal skin, increased IL-2 levels may mediate pruritus; furthermore, serum IL-2 levels are significantly higher in patients with psoriasis than in healthy subjects (76, 77). These findings also shed light on the need to consider IL-2 inhibitors in the design of therapeutic agent for psoriasis, even if abating IL-2 production is not necessarily the primary goal of the psoriasis treatment.

### Salp15 Inhibits the Expression of Cytokines by Dendritic Cells by Interacting With DC-Sign

Dendritic cells (DCs) are known to be the most potent of the antigen-presenting cells. The DCs can activate the initial immune response and have immunomodulatory effects that are related to the multiple pattern recognition receptors (PRRs) they express. The PRRs are composed of five families, including the C-type lectin receptors (CLRs), toll-like receptors (TLRs), RIG-I-like receptors (RLRs), nucleotide-binding oligomerization domain (NOD)-like receptors (NLRs) and DNA sensors (78). Among these receptors, DC-SIGN is a major member of the CLRs that can interact with various pathogens. The activation of DC-SIGN prompts receptor-specific intracellular signals that modulate gene expression of cytokines, chemokines, and costimulatory molecules (79). Usually, DC-SIGN does not modulate the activity of transcription factors alone; rather, it can only activate transcription factors when those factors have already been induced by another receptor, such as TLR (80). The ligands that bind to DC-SIGN are mostly carbohydrate structures (79, 80). A recent study confirmed that Salp15 can specifically bind to DC-SIGN as a direct result of structures created by mannose and galactose. This interaction inhibits the TLR-induced production of the pro-inflammatory cytokines IL-12, IL-6, and TNF-α by DCs and reduces the ability of DCs to activate T lymphocytes (19). Salp15 does not prevent the maturation of DCs, but inhibits the production of IL-12p70, IL-6, and TNF-α in a dose-dependent manner (19).

Cytoplasmic DC-SIGN motifs are known to induce intracellular signaling pathways, although the mechanism underlying these effects have not yet been elucidated. It is currently recognized that activation of the serine/threonine kinase Raf-1 is crucial to intracellular signaling transduction induced by the ligand-binding DC-SIGN (81). Hovius et al. (19) found that silencing Raf-1 in DCs by RNA-interference completely abolishes, Salp15-induced cytokine inhibition. In terms of DC-SIGN signaling upstream of Raf-1, interactions with the active form of Ras can change the conformation of Raf-1, which is a prerequisite for its activation (80). However, the conformational change of Raf-1 is not sufficient for full activation; the phosphorylation of serine 338 (Ser 338) and tyrosine 340/341 (Tyr 340/341) are also required (80, 81). Binding of Salp15 to DC-SIGN induces the activation of Raf-1, which is similar to the signaling transduction effects caused by the binding of mycobacterial ManLAM (19). Nevertheless, the signaling system downstream of Raf-1 can differ and depends on the DC-SIGN-ligand (81). Unlike the signaling observed with other pathogens such as ManLAM, Salp15/DC-SIGN-induced signaling leads to the activation of MEK (MAPKK) after the phosphorylation of Raf-1. The phosphorylation of MEK ultimately activates ERK, and phosphorylated ERK (MAPK) enters the nucleus to initiate the transcription of multiple transcription factors. Interestingly, the signal induced by Salp15/DC-SIGN does not act through ERK, but directly participates in the decay of *IL-6* and *TNF-*α mRNAs, thus impairing nucleosome remodeling in the *IL-12p35* promoter after the activation of MEK (19). In the previous section, we mentioned that Salp15 specifically binds to CD4, directly inhibits TCR-mediated signaling transduction, and regulates the polymerization of cytoskeletal actin to reduce the redistribution of lipid rafts (62, 64). Salp15 binds to DC-SIGN on DCs and may also interact with the CD4 receptor. This is possibly the reason why Salp15/DC-SIGN induces Raf-1 downstream signaling in a manner that differs from that of other pathogens. However, further research is required to test this hypothesis.

Salp15 regulates the production of cytokines by DCs via the Raf-1/MEK signaling pathway at both transcriptional and post-transcriptional levels. The half-life of *IL-6* and *TNF-*α mRNAs are significantly reduced in the presence of Salp15, thus inhibiting production of the pro-inflammatory factors IL-6 and TNF-α by stimulated DCs (19). Further investigations showed that the levels of IL-12p70 are decreased in LPS-activated DCs in the presence of Salp15, although the stability of IL-12p35 mRNA remains unchanged. The rapid remodeling of the nucleosome of the *IL-12p35* promoter in DCs is significantly impaired by Salp15, which is critical for the efficient initiation of transcription (19). Moreover, Hovius et al. (19) observed that LPS-matured DCs, pretreated with Salp15, block the activation and proliferation of T lymphocytes. Collectively, the interaction of Salp15 with DC-SIGN directly modulates the production of cytokines and T cell activation, which is induced by DCs. The properties of Salp15 in modulating human immune responses, especially those of DCs, may provide novel treatment possibilities (82). The DCs are usually present in tissues that make contact with the external environment, such as the skin, nasal mucosa, and intestines. Thus, Salp15 could be a potential drug for topical agents with therapeutic effects on tissues where DCs are concentrated. For instance, blastic plasmacytoid dendritic cell neoplasm (BPDCN) is an extremely rare tumor that has a predilection for the skin. Furthermore, BPDCN is resistant to standard chemotherapies, shows a very poor response to therapy, and has a poor prognosis (83). The regulatory function of Salp15 on DCs and its specific interaction with CD4 molecules make it a potential candidate for the treatment of such rare diseases. In addition, the inhibitory effects of Salp15 on the cytokines, IL-6 and TNF-α, secreted by DCs may have potential applications in RA therapy (84). Salp15 could also be a therapeutic tool against other autoimmune diseases, such as inflammatory bowel disease (85, 86).

### Salp15 Up-regulates the Expression of CD73 in Regulatory T cells

The CD4+ regulatory T cells (Tregs) are a subpopulation of T cells that play an immunosuppressive role. The Tregs play an important role in maintaining autoimmune tolerance and controlling adaptive immune responses. These cells express CD4, CD25, and Foxp3 on their cell surfaces (87). The immunosuppressive effects of Tregs involve various mechanisms that are still not fully understood. Recent studies have revealed that Tregs express two unique ectoenzymes, ectonucleoside triphosphate diphosphohydrolase (CD39), and 5′-ectonucleotidase (CD73), which synergistically generate pericellular adenosine (88, 89). Furthermore, CD39 can catalyze extracellular adenosine triphosphate (ATP) to produce adenosine monophosphate (AMP), while CD73 can hydrolyze 5'-AMP to adenosine (89). Adenosine is known to enhance the levels of cyclic adenosine monophosphate (cAMP) in target cells that express adenosine receptors; this action interferes with the immune response of target cells.

A recent study found that the presence of Salp15 has long-term effects on activated CD4+ T cells *in vivo*, and that Salp15 does not affect the differentiation of CD4+T cells in the absence of polarized cytokines (65). Those authors further observed that the expression levels of *Nt5e*, which encodes CD73, were significantly increased in activated CD4+ T cells treated with Salp15 (65). Subsequently, the expression of CD73 on the surface of activated CD4+ T cells treated with Salp15 were significantly elevated and the levels of adenosine were also increased in those cells (65). Furthermore, the increased expression of CD73 on CD4+ T cells was still detected in the blood of mice 50 days after the induction of graft-versus-host disease (GvHD) (65). Interactions between adenosine and the adenosine receptor lead to the inhibition of effector CD4+ T cell proliferation and reduced cytokine production, thereby exerting anti-inflammatory effects (89, 90). The increase in CD73 expression is responsible, at least in part, for the persistence of Salp15 on CD4+ T cells (65). Therefore, the role of the exonucleolytic peptides CD39 and CD73, and adenosine signaling in pathogenic mechanisms is emerging, and indicates that these peptides represent potential therapeutic targets for a number of clinical situations, including tumors, solid organ transplantation and psoriasis (91–94).

## Salp15 is a Potential Candidate For Various Therapies

Thus far, we have shown that Salp15 can specifically bind to CD4 molecules on the surfaces of T lymphocytes, ultimately affecting TCR-mediated signaling transduction and leading to reduced levels of LCK and ZAP70 phosphorylation. Furthermore, Salp15 can reduce intracellular calcium levels, and therefore prevent the activation and proliferation of CD4 +T cells and impede the production of IL-2. Salp15 also inhibits the expression of CD25 by CD4+T cells and blocks the secretion of various inflammatory cytokines. Other data show that Salp15 can inhibit the activation of CD4+ T cells, both *in vivo* and *in vitro* (4). The inhibitory effects of Salp15 on CD4+ T cells occur in the early stages of activation. *In vivo*, Salp15 has no effect on the responses of T-independent B lymphocytes, such as IgM antibodies. The IgG antibodies produced by B lymphocytes rely on the cooperation of CD4+ T cells; thus, Salp15 can reduce the production of IgG antibodies (4). Owing to these immunomodulatory properties, Salp15 represents a potential candidate for a variety of autoimmune disease treatments.

### Salp15 as a Potential Therapeutic Candidate for Allergic Asthma

Bronchial asthma is a respiratory disease characterized by chronic airway inflammation, airway hyperresponsiveness (AHR), and variable airflow obstruction, which results in persistent eosinophil infiltration, excessive mucus secretion, and subepithelial fibrosis (95, 96). The annual prevalence of asthma is increasing. The World Health Organization data estimate that the number of patients with asthma will increase by 100 million by 2025 (97). Asthma negatively affects the quality of life and creates a significant global economic burden (97). Therefore, research into the treatment of asthma has far-reaching significance. It is now well-established from various studies, that a Th1/Th2 imbalance is the basic pathological feature of asthma, and Th2 cells and the Th1/Th2 imbalance comprise the basic pathophysiological manifestation of asthma (98, 99). The Th2 cells produce a range of pro-inflammatory factors, including IL-4, IL-5, IL-9, and IL-13. These cytokines play an important role in the pathogenesis of asthma, as they can help IgE-producing B cells, eosinophils, mast cells, and basophils to undergo growth, differentiation, and recruitment (98). Previous studies have also verified that the T lymphocyte subset, Th17, is also involved in the pathogenesis of asthma (100). Drugs targeting CD4+ T cells may therefore represent key new discoveries in the treatment of asthma.

Paveglio et al. (21) were the first to confirm that Salp15 has a therapeutic effect on experimental asthma. Those authors sensitized BALB/cJ mice via the intraperitoneal injection of ovalbumin (OVA) in aluminum hydroxide, with and without Salp15, to establish a model of allergic asthma. Aerosolized OVA was then administered to all mice (21). Those authors were surprised to find that the symptoms of allergic asthma in mice treated with Salp15 were significantly reduced compared with the control group. Such symptoms included reductions in the eosinophil count, airway inflammation, mucus secretion, Th2 cytokine production, OVA-specific IgG1 and IgE levels, as well as AHR (21). The specific binding of Salp15 to CD4 inhibits the proliferation and differentiation of CD4+ T cells, and can particularly suppress Th2 and inflammatory cytokines, thus playing a predominant role in the development and prevention of allergic asthma. In fact, both Th2 and Th17 are known to be involved in the pathogenesis of asthma, and are dominant participants in the eosinophilic and neutrophilic phenotypes of asthma, respectively (101). The Th17 can promote neutrophilic inflammation in the development of AHR; such inflammation is related to the severity of asthma (100, 101). However, Juncadella et al. (66) showed that the differentiation of Th17 cells is increased in the presence of Salp15, both *in vivo* and *in vitro*. This may be due to the fact that Salp15 inhibits the production of IL-2, thus interfering with the balance between cytokines and increasing the differentiation of Th17 cells (66). This is probably the mechanism by which Salp15 exerts a therapeutic effect on allergic asthma. Whether Salp15 exerts similar effects in other forms of asthma has not yet been elucidated and requires further research.

### Salp15 as a Potential Therapeutic Candidate for GvHD After Allo-HSCT

Allogeneic hematopoietic stem cell transplantation (allo-HSCT) is considered a curative treatment for many patients with hematological malignancies, immunodeficiencies, and autoimmune diseases. Furthermore, GvHD is a major complication after transplantation (102). Despite significant advances in HSCT over the past 20 years, GvHD remains the leading cause of morbidity and mortality in HSCT recipients after transplantation (103, 104). The GvHD is mainly caused by the immune response of donor T lymphocytes to the recipient cells (which are regarded as foreign antigens). Activated donor T cells recognize recipient antigens, including human leukocyte (HLA); this recognition combined with histocompatibility can cause serious deleterious effects on the recipients (104). Donor CD4+ T cells are known to play a crucial role in the pathophysiology of GvHD, and Tregs are regarded as an inhibitory CD4+ T cell subpopulation (105). Experimental studies of GvHD have demonstrated that Salp15 shows a persistent interaction with CD4 and exerts long-lasting immunosuppressive effects on activating CD4+ T cells (65). In an earlier study (65), splenocytes were extracted from C57BL/6 (H-2b) mice and injected into the peritoneal cavity of CB6F1 (H-2b, d) mice; this was then followed by the intraperitoneal injection of Salp15. Salp15 also protected the kidney from the deposition of immune complexes, a hallmark of chronic GvHD (65). That study indicated the therapeutic potential of Salp15 in GvHD after HSCT, and may be also applicable to GvHD that occurs after other forms of allogeneic transplantation. However, further preclinical experiments are required to test these hypotheses.

### Salp15 Blocks the Binding of HIV-1 gp120 to CD4

Acquired immune deficiency syndrome (AIDS) is one of the most life-threatening diseases worldwide. The etiological agent of AIDS is the human immunodeficiency virus (HIV), an enveloped virus classified in the Retroviridae family. There are three major sub-types of HIV: HIV-1, HIV-2, and HIV-3. Of these, HIV-1 is currently the most prevalent clinical subtype worldwide (106). The gp120 molecule is a glycoprotein found in the envelope of HIV-1, which promotes HIV-1 entry into the host cell and is essential for HIV infection (107). Based on the results of current studies, the infection of mammalian cells by HIV-1 involves three main steps: the binding of gp120 to CD4, which changes the conformation of gp120, which allows the glycoprotein to interact with the chemokine co-receptors, CCR5 or CXCR4, on the surface of the mammalian cell. Eventually, fusion of the viral membrane and the mammalian cell membrane is facilitated by another glycoprotein (gp41), which leads to the release of viral particles into the mammalian cell (106–108).

Interestingly, Salp15 and gp120 are both ligands for CD4 that bind to the D1 domain of sCD4; however, these ligands induce completely different structural changes in the four sCD4 domains (63). Compared with Salp15, HIV-1 gp120 binds to CD4 and changes the internal architecture of sCD4, while Salp15 mainly alters the global structural features of sCD4 (63). Ligand-induced changes to the structural shape of sCD4 are specific and closely related to their biological actions in mammals (63). Because the regions of CD4 that interact with Salp15 and HIV-1 gp120 overlap, the question arises as to whether Salp15 could act as a competitive gp120 blocking agent. Another study (109) gave a positive answer to this hypothesis. *In vitro* microtiter and quantitative fusion assays showed that Salp15 blocks the interaction of gp120 and CD4 in a concentration-dependent manner, and the formation of syncytia by cells expressing gp120 and CD4 is significantly reduced in the presence of Salp15. As mentioned above, the C-terminal portion (P11) of Salp15 is responsible for the binding of CD4. A previous study suggested that Salp15 competes with gp120 for an association with CD4, owing to spatial effects related to the interaction of the entire Salp15 protein with T cells, and not P11 (109). Nevertheless, P11 is able to bind to gp120, which is at least partly responsible for the competitive effect elicited by Salp15 (109). The neutralizing activity of the interaction between Salp15 and HIV-gp120 predicts that this salivary protein may become a new template for the recognition of epitopes in HIV envelope proteins that produce neutralizing antibodies (109). Consequently, Salp15 from ticks has the potential to contribute to the prevention and treatment of AIDS in the future.

One further point to consider is that DC-SIGN on the DC surface can bind to HIV-gp120 with high affinity, thereby facilitating the transport of HIV from the infected site to lymph nodes, and causing the infection of T lymphocytes (110, 111). As reviewed above, Salp15 can also interact with DC-SIGN. Further research is now needed to investigate the specific relationship between Salp15 and gp120.

### Salp15 as a Potential Candidate for Dermatological Treatment

Many autoimmune diseases, such as systemic lupus erythematosus (SLE), are accompanied by skin lesions. Furthermore, SLE is characterized by the deposition of autoantibodies and formation of immune complexes, and can be associated with organ damage mediated by inflammation (112–115). Cutaneous lupus erythematosus (CLE) is one component of SLE manifestation, when the disease is associated only with skin injury. Apoptosis of skin keratinocytes (KCs) plays a significant role in the pathogenesis of SLE skin lesions, and is most likely due to the production of pro-inflammatory cytokines and inflammatory cells (113, 114, 116). The KCs are the staple constituent cells of the epidermis, the main producer of skin chemokines and inflammatory factors, and the physical and immune barrier of the skin. *In vitro* experiments revealed that Salp15 inhibits inflammation produced by human primary keratinocytes in response to *B. burgdorferi* sensu stricto N40 cells or OspC (117). Furthermore, Salp15 not only impedes the expression of monocyte chemoattractant protein 1 (MCP-1), IL-8, and AMPs (hBD-2, hBD-3, RNase 7, and psoriasin) at the mRNA level, but also reduces the concentrations of IL-8 and hBD-2 at the protein level (117). The ability of Salp15 to inhibit the inflammatory response of KCs may therefore be applied to the treatment of lesions caused by autoimmune skin diseases or excessive inflammatory reactions. However, further *in vivo* experiments and preclinical research are necessary to prove these hypotheses.

A critical pathological factor for SLE skin lesions is the deposition of IgG in the skin (115). Other skin diseases, such as bullous pemphigoid (BP), are also associated with the deposition of autoantibodies. In recent years, IgG and IgE antibodies have been found to play a key role in the pathogenesis of BP (118, 119). Because Salp15 can inhibit the proliferation of CD4+ T cells and the production of IL-2, it can also hinder T cell-dependent IgG and IgE production and may serve as a potential target for suitable dermatological treatments.

## Potential Problems

A previous description considered Salp15 as a precursor of treatments for autoimmune diseases, whereas another investigation has shown the opposite effect in murine experimental autoimmune encephalomyelitis (EAE), which mimics multiple sclerosis (66). The occurrence and progression of EAE is associated with myelin-specific CD4+ T cell activation. However, mice treated with Salp15 show increased pathologies upon induction of EAE. The reason may be that Salp15 can promote the activation of Th17 and increase the levels of IL-17 *in vivo*, and also enhance the differentiation of Th17 in the presence of IL-6 and absence of TGF-β *in vitro* to worsen EAE (66). All drugs can have adverse reactions, so we need to maximize their advantages. We can reduce the occurrence of side effects through optimization of drug use. As a biopharmaceutical, Salp15 has special characteristics, including its highly targeted effects, such as species-specificity, immunogenicity, short half-life, and poor stability (120). Almost all protein-based biotherapeutics are unable to avoid immunogenicity, which may eventually lead to lower drug concentrations, reduced efficacy, and an increased risk of adverse reactions. The patient's immune system recognizes biotherapeutics as foreign molecules and produces anti-drug antibodies (ADA), which may affect the safety and effectiveness of the treatment. Although one study has demonstrated that Salp15 is one of the antigens that confers protective effects to mice against Lyme disease (121), no preclinical immunogenicity studies of Salp15 currently exist. However, some biopharmaceutical products have induced the production of ADA, which affected neither the efficacy not the safety of the therapy (122, 123). Some studies show that tick salivary proteins are more suitable as potential pharmacological agents due to their lower levels of cytotoxicity and immunogenicity (124). We could attempt to reduce drug immunogenicity through protein engineering (125). Nevertheless, evaluation of immunogenicity in preclinical and clinical trials is an important consideration in the study of biopharmaceuticals. We need further research for the discovery and approval of suitable therapeutic candidates.

## Future Directions

Disorders of the immune system can lead to a spectrum of diseases, including autoimmune diseases, inflammatory, and even malignant tumors. Current treatments of such diseases are limited, and the effects are not promising, particularly for chemotherapies accompanied by serious adverse reactions. Therefore, scientists now aim to find natural compounds with enhanced physiological activity and novel chemical structures. Natural products have spatial structures that differ from those of chemically synthesized drugs, and also tend to have higher target specificity, affinity, and fewer adverse reactions (126). As the largest and most diverse biological population on earth, arthropods provide a rich source of chemical and biological information for the study of biopharmaceutical compounds. Arthropod-based therapeutics are still in their infancy compared with plant-based or microorganism-based therapeutic agents (127). A variety of biologically active substances derived from tick saliva may be valuable resources for the treatment of various diseases (82). Although Salp15 has not yet advanced to pre-clinical research, its pluripotent effects suggest it may be an attractive candidate for use in immunotherapy. The specificity with which Salp15 binds to CD4+ T cells, and the inhibition of CD4+ T cell activation and proliferation are very promising properties for the treatment of immune diseases. Salp15 not only directly inhibits CD4+ T cell activation, but also plays a key role in immunomodulation by regulating the increased levels of CD73 expression by Tregs. Collectively, Salp15 may play a therapeutic role in a variety of diseases, especially autoimmune diseases. Further research should aim to fully validate these roles and determine the clinical utility of Salp15. Salp15 is a promising candidate for the discovery of new therapeutic agents; however, there is still much to be done for it to go from the bench to the bedside (128).

## Author Contributions

FB, AL, and SW designed the concept and planned the work. FW, ZJ, YP, MJ, YB, GZ, TC, LLu, LLi, ZD, and M-EA took part in the work. SW drafted the manuscript. YP refined the Figure 2. FB and AL revised the manuscript and agree it to submitted.

### Conflict of Interest

The authors declare that the research was conducted in the absence of any commercial or financial relationships that could be construed as a potential conflict of interest.
